# The ACE Brain

**DOI:** 10.3389/fncom.2016.00122

**Published:** 2016-11-25

**Authors:** Massimiliano Zanin, David Papo

**Affiliations:** ^1^Department of Life Sciences, Innaxis Foundation and Research InstituteMadrid, Spain; ^2^Departamento de Engenharia Electrotecnica, Faculdade de Ciencias e Tecnologia, Universidade Nova de LisboaLisboa, Portugal; ^3^GISC and Computational Systems Biology Group, Center for Biomedical Technology, Universidad Politécnica de MadridMadrid, Spain

**Keywords:** complex network theory, functional networks, Boolean modeling, EDVAC, ACE

Good metaphors can help understanding complex systems. The brain-computer metaphor arguably started when McCulloch and Pitts (1943) put together the idea of an artificial computational neuron with multiple inputs, either inhibitory or excitatory, a single branching output and a threshold for firing, proving that in principle a neural network made of these logical neurons could carry out very general computations. This meant that the brain could be treated as a computer and the neuron as its basic switching element. Research in cognitive neuroscience has ever since revealed many important differences between brains and computers, so that the brain-computer metaphor has gone through difficult days.

While appreciating these differences may be crucial to understanding the mechanisms of neural information processing, some of the similarities, at the computational, algorithmic, and implementation levels pointed at in the early days of computer science and artificial intelligence have their value too. Computers and brains share common goals, i.e., to distribute and process information. Moreover, several common organizational features, e.g., how memories are stored at different levels of readiness, and how different computations are performed at different physical locations prompted suggestions that the brain as a highly complex automaton (Searle, 1990; Litt et al., 2006; Istrail and Marcus, 2012), giving rise to a large body of literature aimed at representing the brain as a Boolean system (Baron, 2013). While at micro and meso-scales this respectively involves understanding how neural spikes may encode Boolean information (Rieke, 1999) and describing how individual computations are executed, e.g., finding equivalences between neuron configurations and Boolean operations (Siu and Bruck, 1990; Maass, 1996), at macro-scales an interesting question is the extent to which CPU architectures and brain regions share functionally important features.

Neuroscientists' models of brain functional organization, and in particular of how a given task recruits brain resources, bear important analogies with the way computer elements are arranged and activated to perform complex operations. In modern CPUs, data are distributed across different sub-units by a central controller, a structure inspired by the research performed in the 40s by von Neumann (1993). However, this is not the only possible configuration, and we compare it with the alternative proposed by Alan Turing in the same decade (Carpenter and Doran, 1986). How does the underlying model of computer functioning influence the way neuroscientists describe the brain? For instance, at a system-level of description, neuroscientists typically want to extract the minimum sub-system of the whole brain necessary to execute a given task. Suppose in particular that brain activity is endowed with a network representation (Bullmore and Sporns, 2009). What would the minimal subsystem look like? We propose that Turing's approach is more representative of the human brain, and discuss when functional networks may yield misleading results when applied to such a system.

## EDVAC vs. ACE

Modern computer history starts with two different approaches. In 1945, John von Neumann proposed the first description of the logical design of a computer using the stored-program conception in the famous *First Draft of a Report on the EDVAC*. The ACE (Automatic Computing Engine), the computer designed by Alan Turing, was presented nearly a year later, but, the official chronology notwithstanding, EDVAC was in fact profoundly influenced by ACE (Copeland, 2012). The two approaches share numerous common features, which are the result of the attempt at implementing the same theoretical framework, i.e., the Turing's universal machine. Yet, they also present some important differences in the way information is transmitted between the different computation elements.

**Figure 1:**
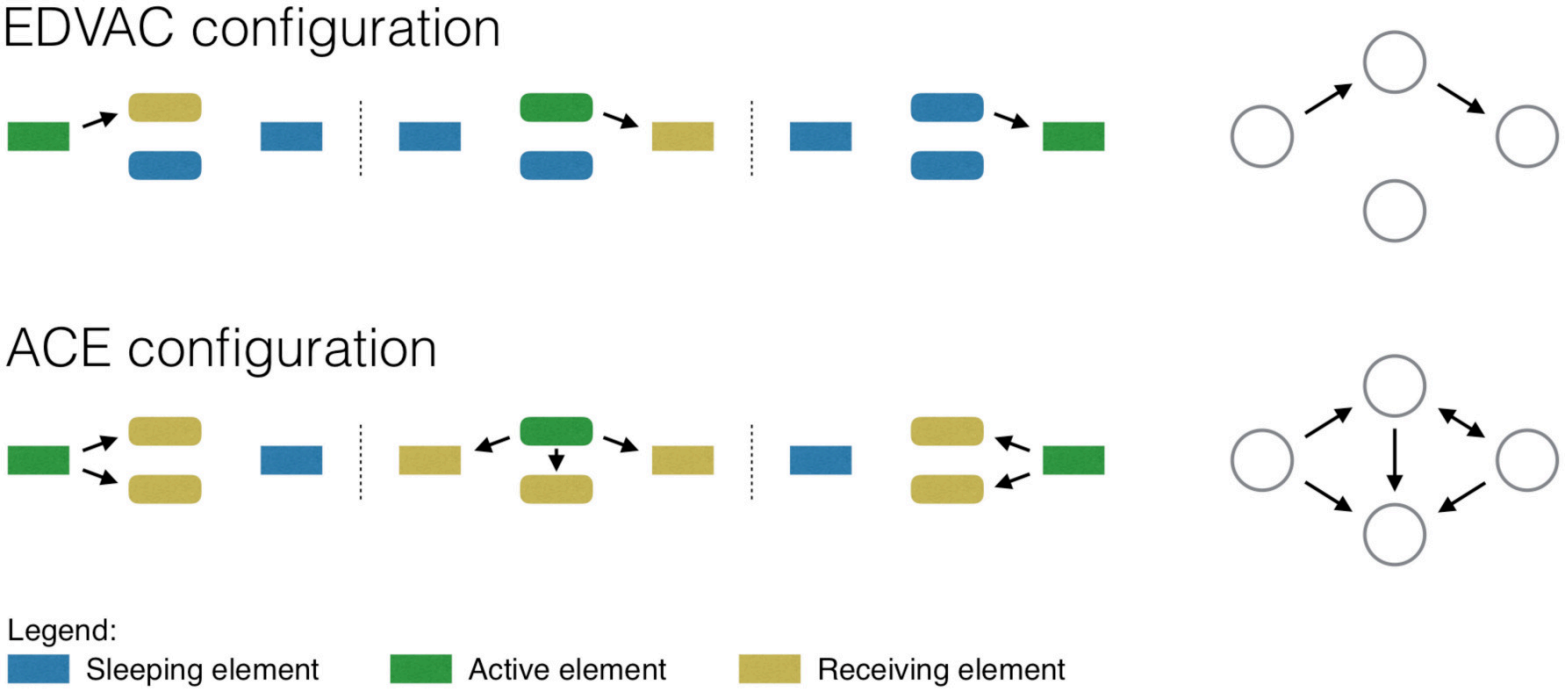
**Representation of a simple computation, as performed by an EDVAC (top) and ACE (bottom) computers**. The two networks on the right depict the resulting functional networks.

The EDVAC configuration includes a central control unit, i.e., an element in charge of defining when each computation element should be activated. Figure 1, top panel, graphically depicts a computation that requires moving information from the left to the right unit. In the first step, the information is stored in the left-most unit; the control unit then activates the central top element, which receives and processes the information; finally, the control unit activates the transfer to the right-most element, which finishes the required computation. Note the efficiency of this approach: At each step of the computation, only those units that are required are activated—for instance, the central bottom unit is never used.

Turing recognized that, in spite of its efficiency, the EDVAC configuration was difficult to implement, as the control unit required a large number of vacuum tubes—which, at the time, were both expensive and unreliable. He thus designed an alternative solution, in which the sequence of operations was only controlled by the movement of information through different memory buffers. In the ACE configuration, at each step of the computation, the input information is shared among all elements, so that all elements receive and compute over it; a memory movement command is then issued, and the result of interest—i.e., the one required for the next step- is again shared. Figure 1, bottom panel, represents such process: Note how, at each step, all elements receive and compute the information processed in the previous time frame.

The EDVAC and ACE configurations thus differ in a very important aspect: While the former requires a specific instruction for each operation (like addition, rest, *etc*.), the latter can theoretically perform all operations with just one command (the information movement one).

## Reconstructing functional networks

Suppose that one does not know how a specific computation is performed, and tries to recover the structure created by information movement through a *functional network* (Bullmore and Sporns, 2009; Papo et al., 2014). Functional networks are created by mapping the elements of the system to nodes, which are pairwise connected when some common dynamics is detected between them. When information is transmitted from one computation unit to another, the internal dynamics of these units becomes coupled, and a corresponding link is created. For the systems depicted in Figure 1, one would expect links to appear between elements that have shared, at some point in time, their information.

How would the EDVAC and ACE configurations be represented by functional networks? In the former, information is sequentially moved between the elements involved in the computation, and this would reflect in the corresponding network. Figure 1, top right panel, depicts the result as an unequivocal feed-forward path. On the other hand, the ACE configuration (bottom right panel) results in a complicated set of connections, in which such path is blurred. The reader familiar with functional network representations of the human brain would recognize Figure 1 bottom right as a typical output of such analyses. Even a simple cognitive task, e.g., one involving a motor response to a specific visual stimulus, results in a network connecting the whole brain, including regions that have *a priori* no relation with the computation being performed (e.g., the auditory cortex). If the brain is understood as an EDVAC system, this means that all brain regions are somehow actively involved in the computation. On the other hand, an ACE-based approach would suggest that this is not necessary: All brain regions are involved in the computation, but only because they all receive and compute external inputs on a continuous basis, irrespective of whether the result is used or not at subsequent steps. Following the previous example, the auditory cortex may receive information from visual areas, as often auditory and visual stimuli would need to be integrated; nevertheless, the result of this computation may not be used under all circumstances.

While the EDVAC configuration is more efficient from a technical perspective, as computational units are activated only when needed, and has indeed been used ever since as the base of modern CPUs, the brain organization resembles rather more that of an ACE device. Specifically, the brain has no centralized control unit, which coordinates how information should be moved between different regions; coordination is decentralized and acts locally. Additionally, the main connections between different brain areas are mostly hardwired; while there exist mechanisms for inhibiting brain regions and connections not needed in a given cognitive task (Desimone and Duncan, 1995; Chawla et al., 1999), such mechanisms are far from being perfect on/off switches. As a result, the functional network representation associated with an ACE system is not strictly isomorphic to that of the underlying hardware, as in an EDVAC one: The dynamics within an ACE can only very partially be accounted for by looking at the physical circuitry in which the dynamics takes place. In this respect, then, an ACE computer more closely resembles a disordered spatially extended system such as the brain than an EDVAC computer and is more consistent with the redundancy and degeneracy characterizing the brain.

## Interpreting functional networks

Most neuroscientists implicitly assume that the brain has an EDVAC-like structure, and therefore use functional networks as a way of extracting the minimum necessary and sufficient sub-system to execute a given task. While it is reasonable to assume an EDVAC structure for sensory tasks, for which the brain evolved dedicated and largely segregated hardware, this metaphor becomes less accurate the more complex the cognitive task. If the brain is more like an ACE, as what we said above seems to suggest, can the minimal information structure still be extracted from functional networks representation of brain activity? As depicted in Figure 1, this is not as straightforward as the EDVAC case, as an ACE generates many indirect connections.

The functional network framework is flexible enough to allow representing both EDVAC and ACE systems, and neutral as to the true nature of the underlying system. One may further hypothesize that the equivalent of the EDVAC network is present within the ACE representation, though masked by noise. Therefore, the neuroscientists' task of extracting the minimum sub-system may be seen as tantamount to extracting an EDVAC backbone from the ACE system, such that only primary information movements (i.e., links) are highlighted. There may be (at least) two possible ways of performing such extraction. As Figure 1 suggests, the only connections really involved in the computation are those that connect the input with the final output. If one was able to exactly define the start and end points of a computation (e.g., in the previously discussed example, respectively the visual and the motor cortex), then it would be possible to isolate only those connections that form a connected path between these two regions. However, the path is hardly ever a single one, as information can be split, processed and then recombined several times. Furthermore, the information seldom travels according to the shortest connectivity path, though this is assumed by most complex network metrics (DeDeo and Krakauer, 2012). Finally, the identification of the starting point is often non-trivial: For instance, information may be retrieved from memory and integrated, even if the task itself may not *a priori* require this. One may also resort to information theoretic measures, to understand to what degree a brain region affects the output of a task. For instance, one may try to assess whether the information stored in the dynamics of a given region is related to the associated behavior, e.g., by means of Mutual Information or similar metrics. If a given computation element only receives information, but does not provide any relevant output, its incoming and outgoing connections can be safely deleted. While this approach is typically used at micro- and meso-scales, e.g., in single-cell studies, it has not yet been applied to the macro-level of functional network reconstruction. In the general case, though, if a backbone does indeed exist its nature is non-trivial and supposes a conceptual framework that is as yet non-standard in system-level cognitive neuroscience.

In conclusion, while complex networks represent a powerful instrument for the analysis of brain dynamics, this does not dispense with interpreting the results that they yield (Papo et al., 2014; Zanin, 2015). In turn, interpretation crucially depends on the computational and algorithmic properties of the underlying model of brain activity. In particular, we argued that an ACE-like structure represents the brain's decentralized information processing structure better than and EDVAC-like one, but that delineating the corresponding network structure necessary and sufficient for the execution of a given task is conceptually more arduous. The extraction of a brain subsystem mediating the execution of a given cognitive task based on the assumption that the underlying structure is EDVAC-like may be an important factor explaining why results of network theoretical analyses of brain activity have not yet the specificity required for clinical applications.

## Author contributions

MZ and DP developed the main ideas and wrote the manuscript.

### Conflict of interest statement

The authors declare that the research was conducted in the absence of any commercial or financial relationships that could be construed as a potential conflict of interest.
